# To be on the safe site – Ungroomed spots on the bee’s body and their importance for pollination

**DOI:** 10.1371/journal.pone.0182522

**Published:** 2017-09-06

**Authors:** Laura Koch, Klaus Lunau, Petra Wester

**Affiliations:** Institute of Sensory Ecology, Heinrich-Heine-University, Düsseldorf, Germany; Indian Institute of Science, INDIA

## Abstract

Flower-visiting bees collect large quantities of pollen to feed their offspring. Pollen deposited in the bees’ transport organs is lost for the flowers’ pollination. It has been hypothesised that specific body areas, bees cannot groom, serve as ‘safe sites’ for pollen transfer between flowers. For the first time, we experimentally demonstrated the position, area and pollen amount of safe sites at the examples of *Apis mellifera* and *Bombus terrestris* by combining artificial contamination of the bees’ body with pine or sunflower pollen and the subsequent bees’ incomplete grooming. We found safe sites on the forehead, the dorsal thorax and waist, and on the dorsal and ventral abdomen of the bees. These areas were less groomed by the bees’ legs. The largest amount of pollen was found on the waist, followed by the dorsal areas of thorax and abdomen. At the example of *Salvia pratensis*, *S*. *officinalis* and *Borago officinalis*, we experimentally demonstrated with fluorescent dye that the flowers’ pollen-sacs and stigma contact identical safe sites. These results confirm that pollen deposition on the bees’ safe sites improves pollen transfer to stigmas of conspecific flowers sti. Future research will demonstrate the importance of safe sites for plant pollination under field conditions.

## Introduction

To enable sexual reproduction, plants normally use animals as pollen vectors, with their flowers mostly offering nectar [1]. Bees, the most important group of pollinators [2,3], visit flowers for nectar, but also collect large amounts of pollen mainly to feed their offspring [4–6]. Contrastingly, pollen is essential for the plants’ reproduction, serving as a vehicle for gametes. Pollen is expensive, and, unlike nectar, pollen supply is strictly limited [7]. As soon as pollen is collected and groomed into the bees’ transport organs, it is mostly lost for the flowers’ pollination [7]. Bees collect pollen actively, e.g. by gathering pollen from anthers with their forelegs or mouthparts, or in a passive way with their body which is groomed from time to time after contamination with pollen [7]. Bees use specific structures (brushes, combs, scrapers) of their legs to groom and collect pollen. After pollen collection, pollen grains are transferred to transport organs which are the crop, the scopae (dense mass of elongated, often branched, birstles) onthe hindlegs or abdomen, or the corbiculae (cavity surrounded by a fringe of bristles) at the tibia of the hindlegs for homeward transportation [8–10]. Pollen situated in the scopae and particularly so in the corbiculae is more or less secure against being stripped off by floral structures including the flowers’ stigma [7,10]. In addition, especially for corbiculate bees (e.g. *Apis* and *Bombus* spp.), pollen is often mixed with liquids, such as regurgitated nectar and saliva, sticking the pollen grains together and improving their attachment to the bees’ pollen basket. Moreover, these admixed liquids reduce pollen germination, resulting in reduced fruit set [11]. Pollen collection by bees is often very effective that less than 1–4% of the flower’s pollen grains reach conspecific stigmas [4,12–16]. The bees’ need for pollen, their sole protein source for provisioning the larval food, and the plants’ need for pollen to ensure sexual reproduction lead to a conflict for pollen between bee-pollinated plants and pollen-collecting bees [7,17].

Consequently, plants benefit from an efficient handling of pollen and should filter out ineffective pollen vectors or pollen thieves and secure large proportions of their pollen for pollination. Flowering plants have evolved several mechanisms to reduce such pollen losses, for example toxic pollen that cannot be digested or mechanical features that prevent pollen collection [15,18–20]. Preventing pollen collection is also be achieved by hiding pollen in poricidal anthers, hiding anthers in the flower, as well as by displaying cryptic or inconspicuous pollen and anthers [21–26]. Another strategy, not mutually exclusive with hidden pollen, is to guide the bees in a specific position on flowers for optimal pollen placement on the bees and likewise for optimal withdrawal of pollen from the stigmas. In this way, the pollen (or most of it) might be deposited on spots of the bees’ body, so-called ‘safe sites’, where the bees cannot see, taste or feel it, or where they are less capable to groom it off, [10,26–29].

Flower-pollinator interactions have been rarely investigated or discussed in view of this fundamental plant-bee conflict of interest for pollen. It has been observed that honeybees have difficulties in grooming some parts of their body, e.g. behind the head, the central dorsal part of their first thoracic segment and the first two abdomen segments [30]. Field observations of flower-visiting bees (*Apis mellifera*, different species of *Bombus* and other genera) at mainly species of Orobanchaceae and Fabaceae have demonstrated that after pollen accumulation on the bees’ bodies by the pollen-sacs) and subsequent grooming, residual patches of pollen patches remain in specific areas of the bees’ body, mainly on the dorsal and ventral midline of the head, thorax and abdomen [31–42]. These patches are restricted to the spots being dusted with pollen by the flowers of the specific species and do not necessarily include all possible safe sites. Therefore, to better identify safe sites and their importance for pollination, it is also necessary to develop experimental settings.

At the example of the European honeybee, *Apis mellifera* Linné 1758 and the Buff-tailed bumblebee, *Bombus terrestris* (Linné 1758) (both Apinae, Apidae), we developed a method to exactly characterise safe sites on the bees’ body via artificial contamination with pollen and subsequent grooming experiments, and quantification of pollen grains in ungroomed spots. This method allows the determination of areas on the bees’ body that are not groomed by the legs as well as the detailed documentation of the potential safe sites’ positions and sizes, and the amount of pollen deposited. Selective contamination experiments enable to verify the existence of areas on the insect’s body that trigger grooming behaviour. Furthermore, we present a simple, but efficient experiment with fluorescent dye as a pollen surrogate to investigate whether the bees’ safe sites are used by flowering plants to ensure safer pollen transfer to the conspecific flowers’ stigma. We demonstrate this at the example of specialised bee-flowers with sophisticated pollen transfer mechanisms (*Salvia* spp.) and a species with rather unspecific pollen release (*Borago officinalis*).

## Materials and methods

The study was conducted from August 2013 to November 2014 and from June to July 2015 (experiments with *Salvia* L. species, Lamiaceae) using successive laboratory colonies of *Bombus terrestris* from Biobest (Westerlo, Belgium) and different colonies of *Apis mellifera* in the Botanical Garden of the Heinrich-Heine-University Düsseldorf, Germany. Only worker bees were used in the experiments.

### Position, size and pollen amount of safe sites

In order to determine the safe sites on the insects’ body, the following procedure was carried out in the laboratory. The insects were placed in transparent plastic jars (3.5 cm diameter, 8.0 cm height) whose floor was covered with pollen and which were sealed with a foam plug. Hand-collected pollen of two plant species was used to exclude differences in the safe sites’ distribution or area on the bees’ body due to different structure of pollen grains: spiny and pollenkitt-covered pollen of insect-pollinated sunflowers, *Helianthus annuus* L., Asteraceae (collected at the farm ‘Gut zur Linden’, Wuppertal, Germany), and smooth, wind-distributed pollen of pines, *Pinus sylvestris* L., Pinaceae [1,43] (collected at the Botanical Garden HHU, Düsseldorf), respectively. Due to the bees’ repeated flight activity, the pollen grains were stirred up, thus contaminating the bee’s body evenly within a few minutes. The contaminated animals were transferred into a clean jar, standing upside down, with the opening downward causing the bees sitting on the foam plug. The plug was replaced from time to time to avoid recontamination with pollen. During 30 minutes, the behaviour of the bees was observed to measure the time of grooming (performing grooming movements) and to detect how grooming was performed and which body parts were groomed and which not. Afterwards, the animals were kept in the fridge for about ten minutes and then freeze-killed (at least 24 hours). The following day, the animals were photographed with a dissecting microscope Leica EZ4D (Leica, Wetzlar, Germany) together with scale paper to enable measuring areas. The area of each of the safe sites (areas with residual pollen) on the animals’ body as well as the area of the whole insect body (exoskeleton including projecting bristles of caput = head, thorax and abdomen) was measured from the photos with the image processing software Fiji [44], calculating from the scale paper. Although the insects have three-dimensional bodies, the plain areas of the photos were not corrected to the third dimension as only percentages are important and as correction factors (ca. 2), calculated for test purposes, were nearly identical for the different body parts and bee species. Only those safe sites were measured whose outlines where clearly visible. As there was no difference in the grooming process and the position or size of the safe sites between pine pollen or sunflower pollen treated animals in both bee species, the sunflower and pine data were merged to document the safe sites. To count pollen grains (only pine pollen samples) in safe sites, pollen was removed from each part of the insects’ bodies with adhesive tape and the tape with the pollen stuck on black cardboard. This procedure was repeated till no pollen remained on the insects. To facilitate pollen removal from the mesosomal waist, first, pollen of all other areas was removed and then the abdomen was separated from the rest of the body for the treatment with tape. The tape on the cardboard was photographed with the Leica EZ4D and the pollen was counted with the image processing software Fiji [44]. After segmentation of pollen from the image background via thresholding, the number of pollen grains was calculated from the pixel number of the total pollen area and the pixel number of different areas with known amount of pollen. The accuracy of this method was verified by checking numerous pollen accumulations of a known number of pollen grains related to a given pixel number.

To confirm the safe sites found, the autofluorescence of sunflower pollen was used. The groomed insects were illuminated with a UV-LED torch (UVG3 Midlight of Labino, Solna, Sweden) in a dark room and photographed with a SLR Nikon D3000 and AF-S DX VR Nikkor 18-55mm lens (Nikon, Tokyo, Japan) in combination with 12 and 20 mm extension rings.

To be able to compare the pollen quantity of a defined area of the bee’s body after contamination and after grooming, several areas of *A*. *mellifera* were selectively contaminated with sunflower pollen. The bees passed a narrow transparent plastic tunnel (14 mm inner diameter) equipped with sunflower pollen-contaminated paintbrush hairs positioned at the top, the bottom, the right side and the left side, respectively (about 6mm extending into the tunnel). After passing the tunnel and being contaminated with pollen, the bees entered a flight cage. As a control, six bees per treatment were caught immediately after the treatment (without having the possibility to groom themselves) and freeze-killed and pollen was removed and counted (method see above). Ten other bee individuals per treatment were kept for one hour in the flight cage in which about 15 minutes grooming activity took place. Afterwards the bees were freeze-killed and pollen was removed and counted.

Statistical analyses were performed with SPSS 22 (IBM, Armonk, NY, USA).

### Triggering grooming behaviour

To detect the insect’s body parts in which grooming is triggered, *B*. *terrestris* individuals were placed in transparent plastic jars sealed with a foam plug. A yellow pipet tip (200μl), filled with pine pollen, was inserted in the centre of the plug till positioned next to the bee. By gently touching the pipette, pollen grains trickled onto specific sites of the bees’ body. The behaviour of the bees, grooming or not, and the body parts groomed, were recorded within 60 seconds. The behaviour of completely unmanipulated bees (without touching) was recorded as a control. 20 bees were tested per body part and the control, altogether 180 animals. Each animal was used only once.

### Interactions with flowers

In order to investigate whether plants use the bees’ safe sites to ensure pollen transfer, the following experiments were carried out in an indoor flight cage at the example of *Salvia pratensis* L. and *Salvia officinalis* L. with laboratory *B*. *terrestris* as well as with *Borago officinalis* L. (Boraginaceae) and managed *A*. *mellifera* caught in the Botanical Garden (HHU Düsseldorf). *S*. *pratensis* and the two bee species occur naturally in the Botanical Garden and surrounding [45–47]. *S*. *officinalis* and *B*. *officinalis* are cultivated in the Botanical Garden, but their distribution area overlaps with that of *B*. *terrestris* and *A*. *mellifera* in the Mediterranean region [45–47]. As the bumble-bees were flower-naïve, they had to be trained to visit *Salvia* flowers in a flight cage. Therefore, surose solution (70% by weight, being more attractive than the *Salvia* nectar) was added into the flower entrances. Bumblebees were released close to the flowers at which the animals quickly detected the sugar solution. When the bees were familiar with the flowers (after a few visits), five bees per plant species were observed visiting a flower with the anthers (here called pollen-sacs) marked with white UV-active luminous pigment (GP280443, Boesner, Witten, Germany). The pigment was added on the open anthers of the stamen exactly where the pollen is presented. After one flower visit the bees were caught and freeze-killed. Prior to the experiment, we made sure that the pigment is a good equivalent for pollen, having very similar transfer characteristics (incl. area of deposition). Five other bees per plant species were treated in the same way, visiting other flowers of the same species with the marked stigma. The dead bees were illuminated with a UV-LED torch and photographed (equipment and method see above). The position and area of the pigment deposited on the bees’ body was compared with the position and area of an average safe site of 10 bumblebees (area was not determined in the *B*. *officinalis*—*A*. *mellifera* experiment). The area of each safe site on the animals’ body was measured from the photos with the image processing software Fiji [44], calculating from the scale paper. To determine the average safe site, all pictures were aligned as to the bees’ body and overlayed. The average safe site contained all areas formed by overlapping of at least five animals. Honeybees had not to be trained as they visited *B*. *officinalis* flowers in the Botanical Garden. Due to the connivent (converging and touching) arrangement around the style, the longitudinally deshiscent (introrse) anthers form a kind of scatter cone causing pollen to be scattered on the insect [48]. As the pollen application is not localised, it could not be imitated with pigment. Thus, in *B*. *officinalis*, only stigma contact was tested with luminous pigment and pollen application onto the bee was observed directly.

## Results

### Position, size and pollen amount of safe sites

**Fig 1:**
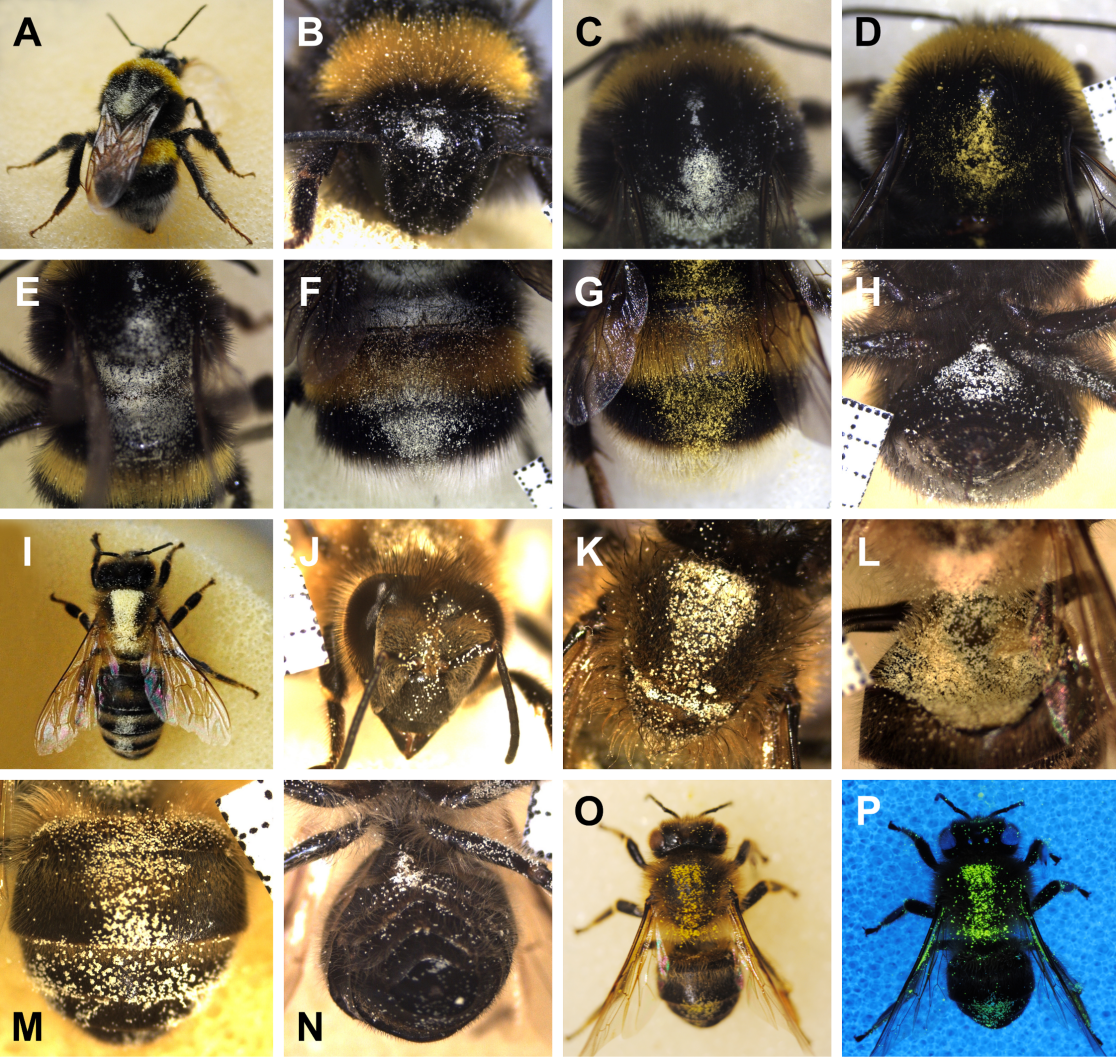
Pollen accumulation in different safe sites of *Bombus terrestris* and *Apis mellifera* after contamination with pollen and subsequent grooming. *B*. *terrestris*: overall view with arrow-shaped safe site on the thorax (**A**), safe site above the insertion of antennae on the head (**B**), dorsal thorax (triangular) (**C**, **D**), waist (**E**), abdomen (**F**, **G**), and ventral abdomen (**H**), *A*. *mellifera*: overall view with broad safe site on the thorax and narrow safe site on the abdomen (**I**), safe site between the eyes (**J**), dorsal thorax (**K**), waist (**L**), and abdomen (**M**), as well as ventral abdomen (**N**), dorsal thorax and abdominal safe site under full spectrum illumination (**O**) and UV-illumination (**P**). All with pine pollen except D, G, O and P with sunflower pollen. Millimetre paper as scale.

In *Bombus terrestris*, areas with residual pollen after grooming were found slightly above the antennae on the head, in a triangular shaped area on the dorsal side of the thorax mainly between the tegulae tapering towards the head, the petiole between thorax and abdomen (waist including propodeum and the first abdominal segment), and the middle area of dorsal part of the abdomen, being wedge-shaped narrowing towards the end of the abdomen (Fig 1A–1H, S1 Video). At the lateral parts of the bee, only few single pollen grains were found, but no accumulations of pollen grains. Some pollen grains were also found on the legs and the ventral side, but larger pollen accumulations were found only on the midline of the proximal ventral abdomen. In *Apis mellifera*, the safe sites were similar to those of *B*. *terrestris*. They were on the head between the antennae and on the dorsal side of the thorax (but mirror-inverted, mainly at the caput-facing end, running as a stripe towards the tegulae and tapering towards the abdomen). Other safe sites were similar to *B*. *terrestris* between the thorax and abdomen, mainly the middle area of dorsal abdomen, and only few pollen grains were found on the legs and the ventral side (here mainly on the abdomen near the thorax side) (Fig 1I–1N, S2 Video). The safe sites found were confirmed by sunflower pollen accumulations visible under UV illumination (Fig 1O and 1P). Autofluorescent sunflower pollen grains were also detected outside the safe sites, but only in very small numbers. Contrary, the pollen accumulations within the safe sites were more clearly visible because of strong autofluorescence.

**Fig 2:**
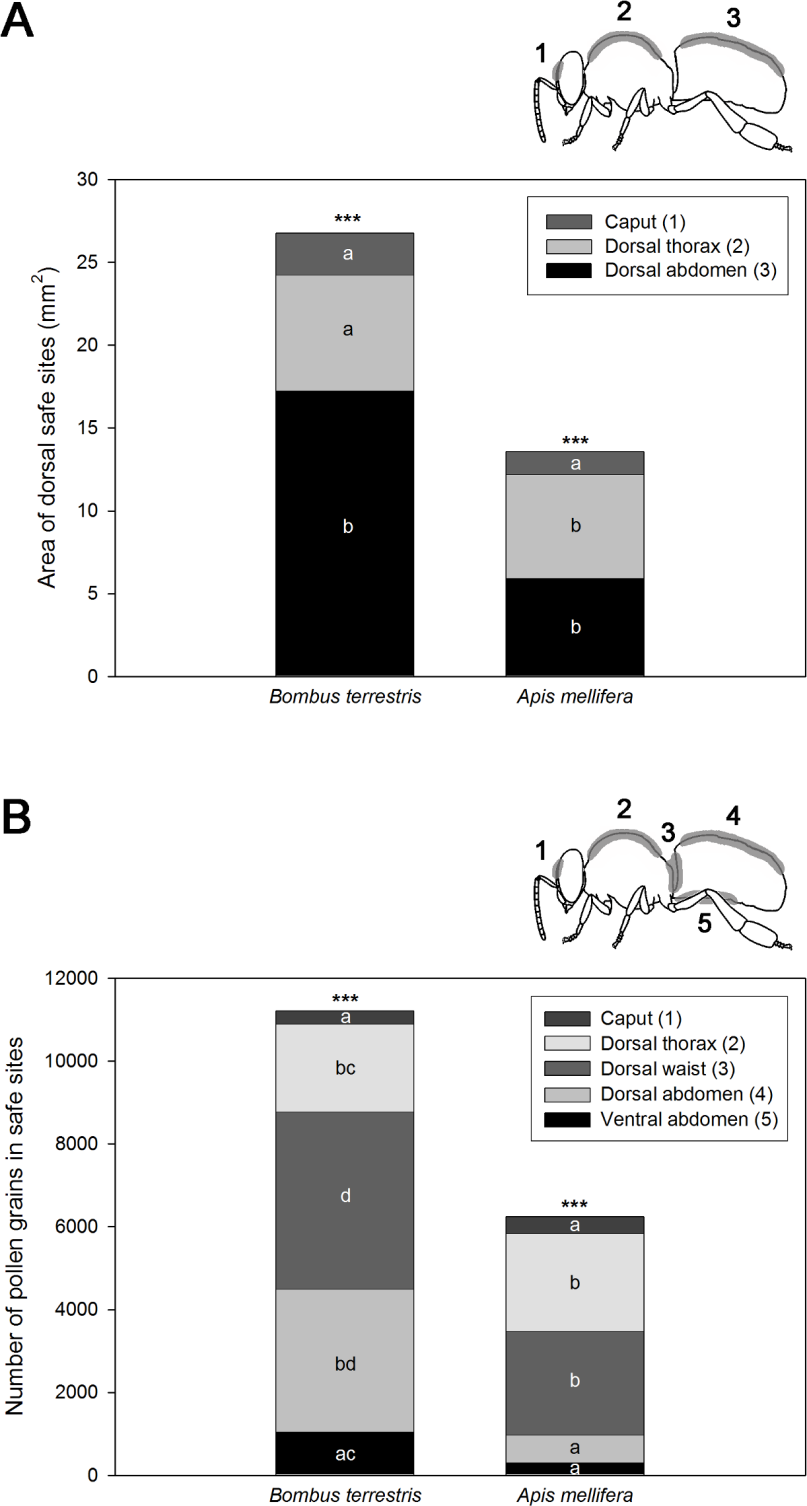
**Dorsal safe site areas (A) and amount of pollen grains in safe sites (B) of *Bombus terrestris* and *Apis mellifera*.** Mean values that share same letters per bee species are not significantly different according to Tukey post-hoc tests at p < 0.05 following ANOVA (*** indicates p < 0.0001). Safe site area (A): *B*. *terrestris*: caput n = 19, thorax n = 32, abdomen n = 17, *A*. *mellifera*: caput n = 14, thorax n = 20, abdomen n = 8. Pollen amount in safe sites (B): *B*. *terresris*: n = 12, *A*. *mellifera*: n = 12.

The dorsal abdomen safe site of the *B*. *terrestris* workers was significantly larger than that of the smaller *A*. *mellifera* workers (Mann-Whitney U-test: p < 0.001, Z = -3.4) whereas the safe sites of the caput and thorax were similar in size in both species (Mann-Whitney U-test: caput: p > 0.2, Z = -1.2, thorax: p > 0.3, Z = -0.94) (Fig 2A, S1 Table). Within the bee species, the safe sites significantly differed in size (ANOVA, *B*. *terrestris*: F = 23.4, p < 0.0001; *A*. *mellifera*: F = 37.6, p < 0.0001). In both species, the head safe site was the smallest one (all p < 0.0001 according to Tukey post-hoc tests, except not significantly smaller than the thorax in *B*. *terrestris*: p = 0.057, Fig 2A). In *B*. *terrestris*, the abdomen safe site was the largest (p < 0.0001 according to Tukey post-hoc tests). Contrary, in *A*. *mellifera*, the thorax and abdomen safe sites were similar in size (p = 0.9 according to Tukey post-hoc test, Fig 2A). The safe sites comprised 24.5% of the dorsal projection of the body in *B*. *terrestris* (caput: 13.4%, thorax: 20.8%, abdomen: 30.2%) and 23.5% in *A*. *mellifera* (caput: 9.7%, thorax: 41.0%, abdomen: 21.1%) (S1 Table).

The amount of pollen grains was significantly larger in the safe sites of *B*. *terrestris* (11208 ± 5779) than in those of *A*. *mellifera* (6240 ± 3135) (Mann-Whitney U-test: p < 0.007, Z = -2.74). The pollen amount of the dorsal waist, dorsal abdomen and ventral safe site was significantly larger in *B*. *terrestris* than in *A*. *mellifera* (waist: p < 0.03, Z = -2.3, dorsal abdomen: p < 0.0001, Z = -4.1, ventral abdomen: p < 0.002, Z = -3.1). There was no significant difference between the number of pollen grains of the head and thorax safe sites (caput: p > 0.2, Z = -1.1, thorax: p > 0.4, Z = -0.9) (Fig 2B, S2 Table).

The number of pollen grains left in the safe sites significantly differed within the bee species (ANOVA, *B*. *terrestris*: F = 13.96, p < 0.0001; *A*. *mellifera*: F = 12.16, p < 0.0001). The dorsal waist contained the most pollen grains, followed by the dorsal abdomen (*B*. *terrestris*) and dorsal thorax (*A*. *mellifera*) safe sites (Fig 2B). Comparing the number of pollen grains on the honeybees’ body before and after grooming shows that nearly all pollen (99.1%) was removed from the lateral parts (remaining pollen on the right side: 0.7%, left side: 1.1%; Mann-Whitney U-test: p = 0.000003, Z = -4.67), but that 19.6% of pollen was left in the safe sites on the dorsal side (Mann-Whitney U-test: p = 0.0005, Z = -3.15) and 15.5% in the safe sites on the ventral side (Mann-Whitney U-test: p = 0.0005, Z = 3.15; for all: before grooming: n = 6, after grooming: n = 10) of the bee (S3 Table).

During the grooming process, the bees wiped their head and antennae with the forelegs without reaching the area between their antennae. With their middle legs, the bees cleaned the dorsal and lateral thorax, except the dorsal area between the tegulae (insertions of wings) in *Bombus terrestris* and a large area right behind the head in *Apis mellifera*. The forelegs and middle legs completely wiped the ventral thorax. The hindlegs groomed the abdomen, thereby removing pollen mainly from the lateral part of the abdomen, whereas the bees could not reach pollen in the middle of the dorsal abdomen. The bees used their hindlegs to clean the wings and removed pollen left at the hindlegs by rubbing the legs against each other or by transferring pollen into the corbiculae (S1 Video, S2 Video).

The time actually spent for grooming was longer when contaminated with sunflower pollen as compared to contamination with pine pollen (significantly longer in *Bombus terrestris*: pine pollen: 4.5 ± 2.8 min, n = 29, sunflower pollen: 8.3 ± 5.5 min, n = 15, Mann-Whitney U-test: p < 0.004, Z = -3.0; not significantly longer in *Apis mellifera*: pine pollen: 10.5 ± 7.1 min, n = 17, sunflower pollen: 15.2 ± 7.8 min, n = 14, Mann-Whitney U-test: p = 0.06, Z = -1.9). Despite the above mentioned difference in grooming times, in both bee species there was neither a difference in the grooming process nor the position or size of the safe sites between the two tested pollen types (Mann-Whitney U-test: *B*. *terrestris*: p > 0.06, Z = -1.9, *A*. *mellifera*: p > 0.5, Z = -0.7). The overall grooming time was longer in *A*. *mellifera* than in *B*. *terrestris* (Mann-Whitney U-test: p < 0.001, Z = -4.3) (S4 Table).

### Triggering grooming behaviour

**Fig 3:**
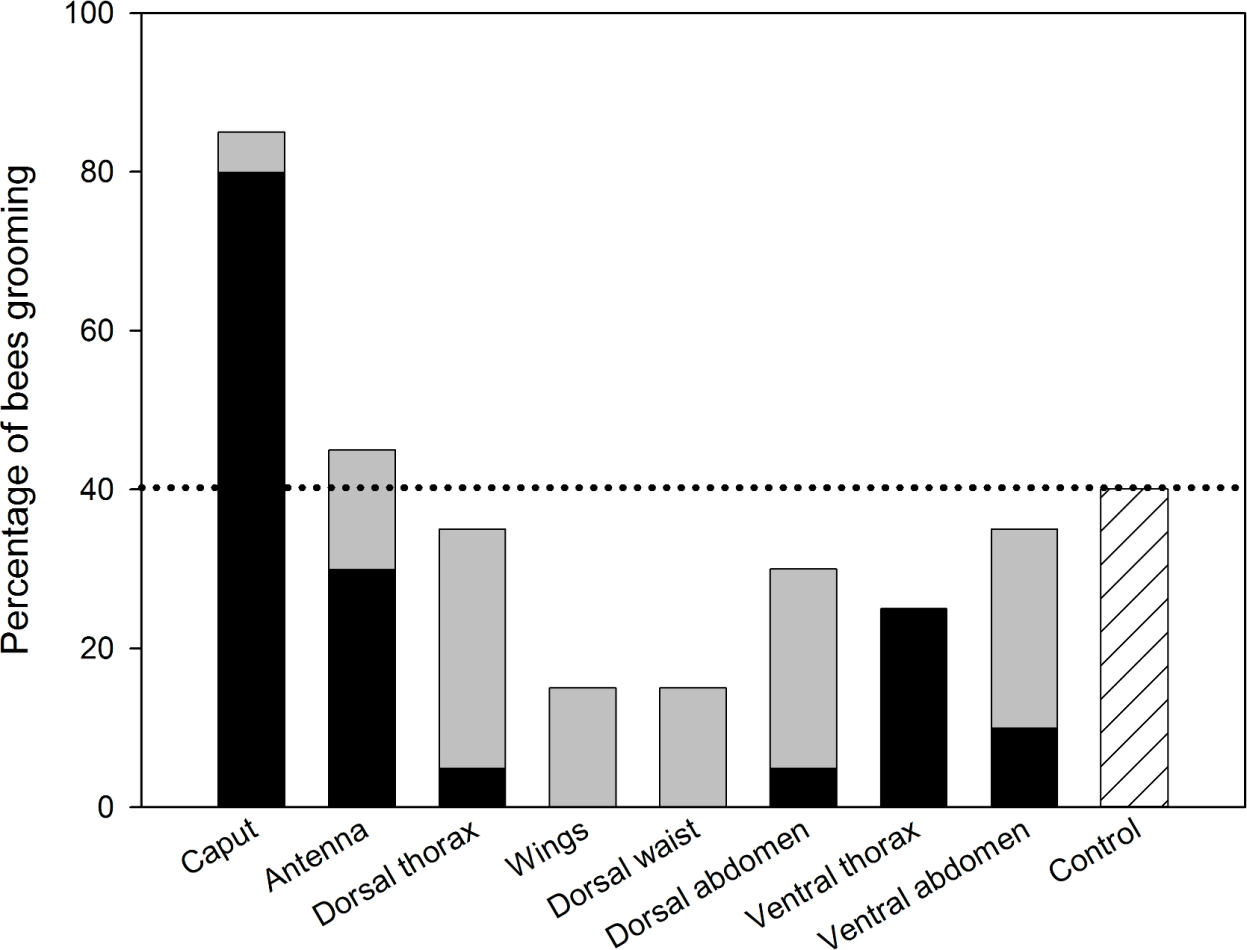
Triggering grooming behaviour of *Bombus terrestris* after contamination of different body parts with pine pollen. Percentage of bee individuals (n = 20 bees per body part and control) showing grooming or wing flapping behaviour including responses at the stimulated body parts (black) and at unstimulated body parts (grey). Control animals were not stimulated.

When pollen contamination targeted the bumblebees’ head, 80% of the bees responded with grooming their head and only very few bees cleaned other body parts (Fig 3, S5 Table). Pollen application on all other body parts resulted in relatively low grooming responses of the contaminated area and other body parts as compared to the control animals that had not been contaminated with pollen. Altogether, the grooming behaviour at the head after corresponding triggering was significantly higher than that at other stimulated body parts in the tested bees and in the control (Fisher’s exact test: p < 0.02).

### Interactions with flowers

**Fig 4:**
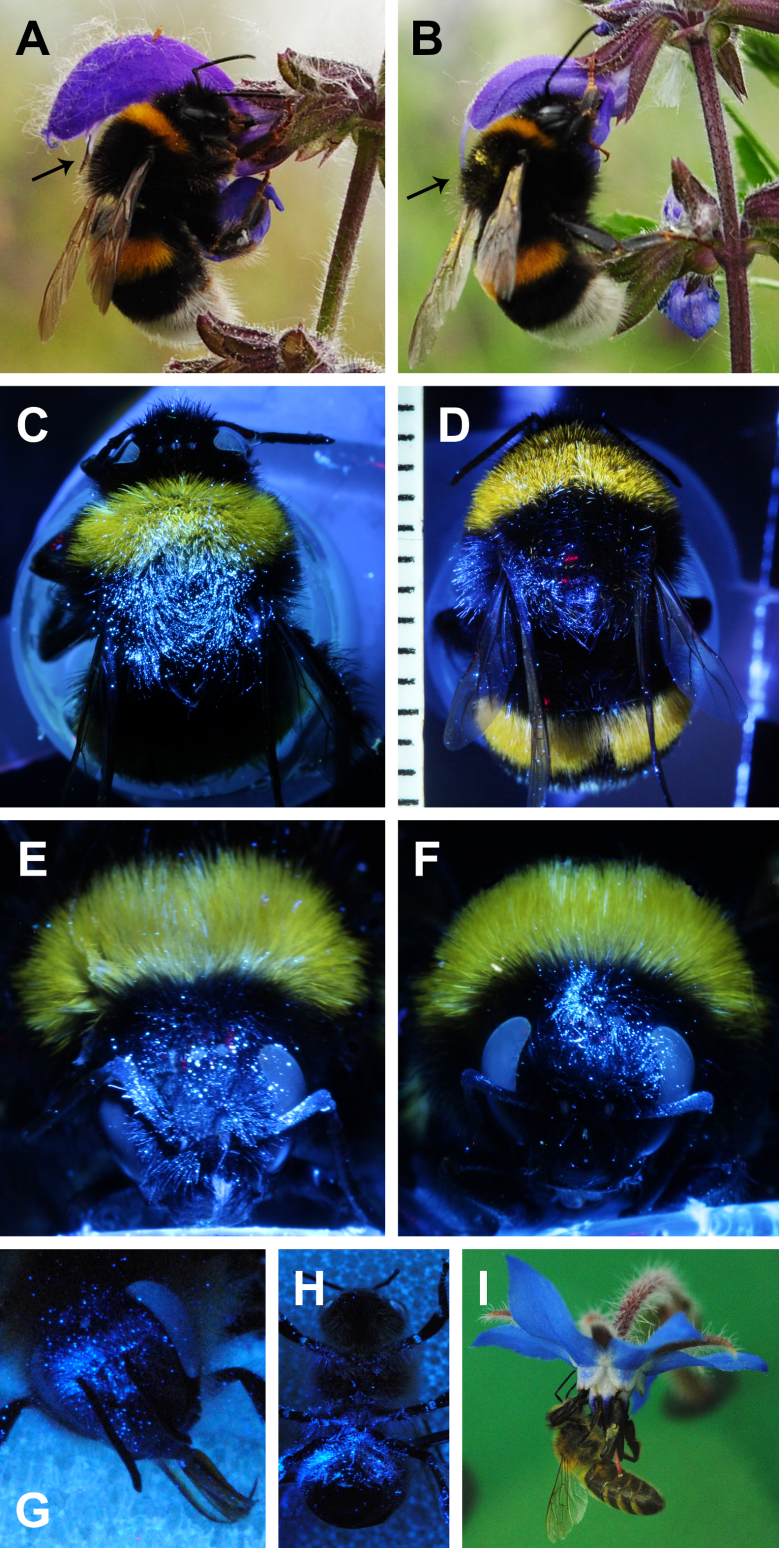
Pollen transfer simulation with luminous pigment on *Bombus terrestris* and *Apis mellifera*. *Salvia pratensis* flower visited by *B*. *terrestris* with the dorsal thorax touching the pollen-sacs (see arrow) (A) and the stigma (see arrow) (B), *B*. *terrestris* under UV-illumination with luminous pigment on the thorax deposited by the pollen-sacs (C) and the stigma (D) of *Salvia pratensis*, and on the head deposited by the pollen-sacs (E) and the stigma (F) of *S*. *officinalis*. *A*. *mellifera* under UV-illumination with luminous pigment deposited by the stigma of *Borago officinalis* on the head (G) and the ventral abdomen (H), *A*. *mellifera* visiting a *Borago officinalis* flower with the stigma of the red style touching the ventral abdomen and the anthers situated above the ventral abdomen (I). Photos C-D: Sofia Marazopoulou, E-F: Leopold Flasch.

After flower visits of *B*. *terrestris* workers to *Salvia* flowers, the luminous pigment was transferred from the pollen-sacs (upper ones in *S*. *pratensis*, lower ones in *S*. *officinalis*) and the stigma exclusively onto the thorax (*S*. *pratensis*) and the head (*S*. *officinalis*) (Fig 4A–4F). In all cases, the transfer took place on the safe sites. 46% of the head safe site area in *B*. *terrestris* is covered by pigment from *S*. *officinalis* pollen-sacs and 37% with pigment from stigmas. In *S*. *pratensis*, 20% of the safe site area on the bumblebees’ thorax was contaminated by the pollen-sacs and 9% by the stigmas. 30% of the total dorsal area on the bee (head or thorax, respectively) covered by pigment from the pollen-sacs is congruent with the safe site, and 29% or 33% of the total area covered by pigment from stigmas is consistent with the safe site of *S*. *officinalis* or *S*. *pratensis*, respectively. In the safe sites, in all cases, the area contaminated with luminous pigment from the pollen-sacs overlapped with that contaminated with pigment from the stigmas. Also in *Borago officinalis* the pigment was transferred from the stigma into the safe sites of the head and the ventral abdomen of *A*. *mellifera*, whereas pollen was deposited allover the ventral side (Fig 4G–4I).

## Discussion

With the combination of experimental contamination of bees with pollen and subsequent grooming by the bees we have been able to precisely characterise safe sites on the bees’ body. The grooming behaviour and the resulting position of the safe sites on *B*. *terrestris* and *A*. *mellifera* were very similar and also independently from the pollen type (*Pinus*, Sunflower). Some of the safe sites in *B*. *terrestris* and *A*. *mellifera* only slightly differ in shape or size. This could either be caused by less effective or shorter grooming behaviour in *B*. *terrestris*, or, alternatively by the different accessibility of the relevant safe sites due to differences in the hairiness and mobility of legs and body.

In both bee species, the dorsal waist area of the mesosoma contained most pollen, followed by the thorax and abdomen, whereas the head and ventral side of the bees had less residual pollen. While the disposition to grooming might only explain the superior grooming activity on the head (probably due to pollen on the eyes blocking the bumblebees’ view), the distribution of pollen in safe sites is rather explainable by their different accessibility by the legs [8]. Thus, grooming of the other body parts might rather aim at transferring the pollen into the corbiculae for homeward transport than being a response to the stimulus of pollen deposition. It is not surprising that the waist area, which is less accessible by the legs, contains the largest number of pollen grains and that the flanks of the bees, which are easily accessible by the legs, lack any safe sites.

To our knowledge, there is no published experimental evidence for safe sites on bees. The identification of safe sites is restricted to the description of patches of pollen grains remaining after the bees’ grooming behaviour following pollen deposition by pollen-sacs during flower visits. The safe sites of *A*. *mellifera* identified in our study are similar to those on the dorsal and ventral midline of honeybees pollinating different *Pedicularis* L. species (Orobanchaceae) in China [38] (S6 Table), indicating the safe sites’ relevance in a natural context. The non-groomed body areas on the dorsal and ventral midline identified here for *B*. *terrestris* correspond to the areas found on other *Bombus* species in China and North America visiting different plants [32–35,38,41] (S6 Table). Similar safe sites have been observed on *Xylocopa* sp. (dorsal thorax) [26], *Megachile ericetorum* (dorsal thorax) [48] and *Euglossa imperialis* (anterior face and between thorax and abdomen) [42] (S6 Table). Safe sites on body areas not found in our study are the cervical groove (neck) (*Bombus* spp.) [36,37,49], the proboscidial fossa (groove on the underside of the head into which the proboscis folds) and other parts of the ventral head (*Bombus* spp., *Anthophora*, *Eucera*, diverse Euglossini and other bees) [31,40,50,51] and the area between the head and the thorax (*Bombus* spp., *Anthophora*) [40], the ventral thorax (*Bombus* spp. and other bees) [31,36,37], the gular region of the head (throat) (*Euglossa imperialis*) [42], the ventral head (*Habropoda laboriosa*) [39] and the incision between head and thorax (*Xylocopa violacea*) [52] (S6 Table). The discrepancies between these areas and the safe sites found in our study might be due to different grooming behaviour/capabilities in the different bee species.

Thus, in general, residual pollen accumulates mainly on the dorsal and ventral bees’ midlines, where the bees probably do not groom at all, groom only less intense, or later in the course of the grooming procedure. With fluorescent dye we could show that the relevant safe sites of *Salvia pratensis* and *S*. *officinalis* were used by the pollen-sacs for pollen deposition and also by the stigma for pollen uptake similar to the observations in the studies mentioned above [31,32,34–40, 50, 52]. This also applies to the transfer of pollen surrogate in *Borago officinalis*. With the complex staminal lever mechanism of thespecialised bee-flowers *S*. *pratensis* and *S*. *officinalis*, pollen would be deposited rather precisely onto the safe sites of *B*. *terrestris*. In contrast, due to the scatter cone mechanism, pollen of *B*. *officinalis* was transferred less precisely onto *A*. *mellifera*. Thus, in *B*. *officinalis* more pollen might be wasted due to the bees’ grooming action.

The larger the congruence between the area contaminated with pollen by the pollen-sacs or that has been contacted by the stigma and the respective safe site of the corresponding pollinator, the lower is the probability of losing pollen according to the bees’ grooming activity. Less pollen loss results in increased pollination efficiency [26].

The safe sites of *B*. *terrestris* are larger and contain more pollen than those of *A*. *mellifera* resulting in an increased probability to get in contact with the pollen-sacs or stigmas. However, other features determining pollination efficiency are the bees’ motivation to collect pollen or groom pollen into the corbiculae (including thoroughness and frequency of grooming). In addition, the bees’ pollen collecting behaviour and morphology of grooming and storing devices affect the number of pollen grains left over for pollination. Also the morphological fit between flowers and visitors (size, shape, functionality, location where pollen gets deposited or stigma collects pollen) as well as the visitors’ physical and mental capabilities to trigger potential floral mechanisms might influence pollen transfer. The ability of flowers to manoeuvre the bees into specific positions on the flower for precise pollen transfer might play a key role for the precision of pollen transfer within safe sites.

In general, since safe sites are on the midline of the bees’ body, specialised, often zygomorphic bee flowers, usually place pollen specifically on the dorsal (nototriby, e.g. in labiate flowers such as many Labiatae including most *Salvia* species) or ventral (sternotriby, e.g. in keel flowers such as many Fabaceae) side of the bee [25,26]. The preferred distribution of the safe sites on the midline of the insects’ body explains why only few bee-pollinated plant species, often with asymmetric flowers, place pollen on the lateral side of the bee (pleurotriby, see also [53]), which is more easily to reach by the grooming legs. Less pollen loss resulting in increased pollination efficiency enables the reduction of costly pollen production and of the number of anthers/thecae (e.g. two thecae per flower in *Salvia* or even only one theca in *Canna* L., Cannaceae) [26].

Altogether, we have experimentally demonstrated safe sites on the bees’ body and their usability by plants to improve safe transportation of pollen grains for pollination. To be able to evaluate the ‘safety’ of the safe sites, experiments should be carried out under field conditions, in which the flowers’ and bees’ shapes and sizes vary and pollen collection and grooming might be influenced by a variety of factors. Then the percentage of saved pollen grains in safe sites should be calculated in relation to the total pollen amount transferred within a flower visit to estimate the importance of pollen transfer in safe sites. This includes pollen that is removed or collected by the bee via grooming and the pollen that gets lost e.g. during flight activity or touching non-stigmatic floral parts or stigmas of interspecific flowers. Additionally, as bees and bumble bees are said to usually groom during flight between flowers [16,41], it should be examined whether grooming behaviour–and thus pollen distribution in safe sites–differs when bees groom during flight as our study is mainly limited to grooming during perching. Also, as grooming activity might differ in bees completely or not fully covered with pollen as in our experiment, the grooming behaviour–and pollen distribution in safe sites–of flower visiting bees (possibly only partially covered) should be examined. It also should be verified whether there are differences in grooming activity and pollen deposition in safe sites of bees searching for nectar as well as in those of bees actively collecting pollen. Moreover, the importance of safe sites could be evaluated by measuring the proportion of safe site pollen delivered onto a conspecific stigma of one or more flowers as compared to the amount of pollen from outside the safe sites. However, pollination is a dynamic process in space and time. Success of pollination and fertilisation is influenced not only by the amount of pollen transferred to a stigma of a conspecific flower [54–57], but also by the time the pollen is exposed to temperature, humidity and UV-radiation, and other factors reducing the ability of pollen grains’ germination [58,59]. The dynamics of pollen turnover in safe sites due to subsequent flower visits, causing both pollen export to stigmata and pollen import onto the bees’ body [29,60], might affect the outcrossing distance [28, 61]. Additionally, the shape, stiffness and position of the style and stigma could determine whether outer (distal) pollen, probably deposited recently, or pollen from lower (proximal) layers, probably deposited earlier, of the safe sites gets transferred [29,62]. Beside the quality of conspecific pollen (e.g. outcross pollen), also interspecific pollen, which might clog the stigma and thus prevent attachment of conspecific pollen, affects the success of pollination [63]. Thus, the study of safe sites on the bees’ bodies and the pollen grains’ ride in space and time and their role in pollination still remains a challenge.

The concept of safe sites for pollen transfer on bees’ bodies does not put the major role of bees as pollinators into question, but might focus future research to guidance of bees on flowers to take favorable positions for safe pollen transport by means of floral filtering of suitable pollinators [26], morphological match between pollinators and flowers [54] and floral guides [55] as well as potential strategies of flower visitors to bypass the flowers’ restrictions (e.g. bees with specialised hairs for pollen collection from specialised bee-flowers) [26] or strategies of plants via shifting to non-pollen collecting pollinators (e.g. birds) [56–57,64–66].

## Supporting information

S1 Video *Apis mellifera*, grooming after being dusted with pine pollen. Note the resulting safe sites on the caput, dorsal thorax and abdomen.
(MP4)

S2 Video *Bombus terrestris*, grooming after being dusted with pine pollen. Note the resulting safe sites on the rear dorsal thorax.
(MP4)

S1 Table Dorsal safe site areas and total body part areas of *Bombus terrestris* and *Apis mellifera* (raw data, and mean ± s.d.). (DOCX)

S2 Table Pollen grain amount in safe sites of *Bombus terrestris* and *Apis mellifera* (raw data, and mean ± s.d.). (DOCX)

S3 Table Pollen grain amount of different body parts of *Apis mellifera* before and after grooming (raw data, and mean ± s.d.). (DOCX)

S4 Table Time [min] spent grooming sunflower pollen and pine pollen of *Apis mellifera* and *Bombus terrestris* (mean ± s.d.). (DOCX)

S5 Table Amount of triggered grooming activities at the stimulated body parts and at unstimulated body parts of *Bombus terrestris*. n = 20 bees per body part and control. Control animals were not stimulated.
(DOCX)

S6 Table Location of safe sites on the bees’ body after grooming behaviour following pollen contamination by pollen-sacs during visits of different plant species. The sites are similar (s) to the location found in this study (*Apis mellifera*, *Bombus terrestris*) or different (d). References given in main text.
(DOCX)
