# Supplementary material for: To be on the safe site – Ungroomed spots on the bee’s body and their importance for pollination
Source: PLoS One. 2017 Sep 6;12(9):e0182522. doi: 10.1371/journal.pone.0182522 (PMC5587100; doi:10.1371/journal.pone.0182522)
Supplement: S1 Table — (DOCX) [file pone.0182522.s003.docx]

**Supporting information**

**To be on the safe site – ungroomed spots on the bee’s body and their importance for pollination**

Laura Koch, Klaus Lunau & Petra Wester*

**S1 Table. Dorsal safe site areas and total body part areas of *Bombus terrestris* and *Apis mellifera* (raw data, and mean ± s.d.).**

| **Safe site area (mm^2^)** | | | | | |
| --- | --- | --- | --- | --- | --- |
| ***Bombus terrestris*** | | | ***Apis mellifera*** | | |
| Caput | Thorax | Abdomen | Caput | Thorax | Abdomen |
| 6.62 | 3.60 | 40.72 | 0.87 | 8.58 | 6.73 |
| 6.19 | 7.90 | 29.91 | 0.14 | 4.50 | 10.72 |
| 5.00 | 13.64 | 27.38 | 1.11 | 8.17 | 4.11 |
| 4.87 | 19.5 | 27.37 | 2.44 | 7.10 | 8.62 |
| 4.42 | 13.94 | 23.61 | 2.63 | 4.35 | 5.51 |
| 3.64 | 2.65 | 20.61 | 1.95 | 4.46 | 5.13 |
| 3.63 | 1.11 | 15.57 | 1.75 | 5.53 | 4.76 |
| 2.30 | 2.61 | 14.25 | 1.81 | 7.67 | 1.89 |
| 2.21 | 0.30 | 14.03 | 0.59 | 4.42 |  |
| 1.96 | 2.98 | 13.97 | 1.87 | 6.52 |  |
| 1.51 | 4.66 | 12.50 | 0.59 | 5.53 |  |
| 1.42 | 16.78 | 12.39 | 2.17 | 8.94 |  |
| 1.12 | 5.83 | 10.99 | 0.80 | 5.11 |  |
| 0.87 | 6.70 | 8.08 | 0.81 | 4.13 |  |
| 0.78 | 1.35 | 7.78 |  | 8.47 |  |
| 0.71 | 2.83 | 7.29 |  | 7.45 |  |
| 0.30 | 2.69 | 6.38 |  | 4.45 |  |
| 0.14 | 1.11 |  |  | 6.65 |  |
| 0.05 | 10.42 |  |  | 6.24 |  |
|  | 1.75 |  |  | 6.44 |  |
|  | 6.47 |  |  |  |  |
|  | 0.53 |  |  |  |  |
|  | 12.61 |  |  |  |  |
|  | 5.27 |  |  |  |  |
|  | 4.39 |  |  |  |  |
|  | 1.59 |  |  |  |  |
|  | 19.19 |  |  |  |  |
|  | 9.71 |  |  |  |  |
|  | 9.74 |  |  |  |  |
|  | 25.36 |  |  |  |  |
|  | 4.64 |  |  |  |  |
|  | 1.98 |  |  |  |  |
| **2.51 ± 2.09** | **7.00 ± 6.43** | **17.23 ± 9.61** | **1.40 ± 0.78** | **6.24 ± 1.61** | **5.93 ± 2.74** |

| **Total body part area (mm^2^)** | | | | | |
| --- | --- | --- | --- | --- | --- |
| ***Bombus terrestris*** | | | ***Apis mellifera*** | | |
| Caput | Thorax | Abdomen | Caput | Thorax | Abdomen |
| 22.23 | 35.83 | 69.43 | 13.99 | 15.64 | 27.39 |
| 27.34 | 40.84 | 41.31 | 15.66 | 16.22 | 23.81 |
| 23.69 | 34.87 | 42.09 | 13.98 | 15.16 | 26.26 |
| 20.96 | 31.40 | 63.35 | 16.61 | 14.84 | 19.65 |
| 17.96 | 27.85 | 61.85 | 14.87 | 12.50 | 20.06 |
| 14.98 | 21.86 | 41.05 | 15.90 | 17.44 | 28.17 |
| 20.41 | 42.72 | 67.74 | 14.82 | 14.14 | 34.07 |
| 18.11 | 23.87 | 47.04 | 16.19 | 14.29 | 33.78 |
| 15.13 | 24.32 | 62.30 | 14.48 | 15.69 | 32.36 |
| 24.04 | 25.53 | 49.94 | 14.62 | 16.15 | 27.25 |
| 16.53 | 42.66 | 72.89 | 18.71 | 16.65 | 32.60 |
| 11.13 | 28.80 | 84.01 | 15.81 | 18.29 | 33.80 |
| 17.47 | 41.29 | 46.38 | 13.72 | 14.40 | 25.98 |
| 15.86 | 27.84 | 34.99 | 13.30 | 16.49 | 33.11 |
| 25.34 | 26.83 | 37.22 | 13.40 | 16.84 | 20.29 |
| 23.72 | 42.51 | 69.32 | 15.08 | 16,63 | 25.68 |
| 17.89 | 33.98 | 67.78 | 13.13 | 12.99 | 33.98 |
| 16.04 | 27.48 | 66.67 | 13.18 | 13.29 | 23.02 |
| 16.63 | 29.53 |  | 15.11 | 15.26 | 28.30 |
| 13.25 | 49.94 |  | 12.69 | 14.48 | 28.33 |
| 15.35 | 34.56 |  | 13.59 | 14.39 | 27.63 |
| 15.96 | 41.33 |  | 13.64 | 14.35 | 26.25 |
| 19.00 | 28.98 |  | 13.18 | 15.46 | 26.77 |
| 20.17 | 28.18 |  | 12.66 | 13.81 | 25.20 |
|  | 33.62 |  | 12.44 |  | 30.02 |
|  | 43.66 |  |  |  | 28.15 |
|  | 29.35 |  |  |  |  |
|  | 40.56 |  |  |  |  |
|  | 33.53 |  |  |  |  |
|  | 34.93 |  |  |  |  |
| **18.72 ± 4.06** | **33.62 ± 7.16** | **56.96 ± 14.48** | **14.43 ± 1.48** | **15.22 ± 1.44** | **28.15 ± 4.72** |
