# Supplementary material for: To be on the safe site – Ungroomed spots on the bee’s body and their importance for pollination
Source: PLoS One. 2017 Sep 6;12(9):e0182522. doi: 10.1371/journal.pone.0182522 (PMC5587100; doi:10.1371/journal.pone.0182522)
Supplement: S2 Table — (DOCX) [file pone.0182522.s004.docx]

**Supporting information**

**To be on the safe site – ungroomed spots on the bee’s body and their importance for pollination**

Laura Koch, Klaus Lunau & Petra Wester*

**S2 Table. Pollen grain amount in safe sites of *Bombus terrestris* and *Apis mellifera* (raw data, and mean ± s.d.).**

| ***Bombus terrestris*** | | | | |
| --- | --- | --- | --- | --- |
| **Dorsal caput** | **Dorsal thorax** | **Dorsal waist** | **Dorsal abdomen** | **Ventral abdomen** |
| 588 | 1935 | 2056 | 2160 | 1806 |
| 96 | 3896 | 4953 | 5901 | 2588 |
| 1335 | 5878 | 6034 | 7962 | 3375 |
| 96 | 2690 | 3310 | 3311 | 906 |
| 32 | 901 | 4925 | 2023 | 259 |
| 463 | 1022 | 2049 | 1301 | 460 |
| 24 | 621 | 2280 | 2187 | 975 |
| 37 | 1138 | 2776 | 1964 | 199 |
| 505 | 859 | 7917 | 3183 | 470 |
| 44 | 1661 | 4580 | 3265 | 548 |
| 51 | 578 | 4426 | 2543 | 526 |
| 567 | 4309 | 5966 | 5505 | 482 |
| **320 ± 395** | **2124 ± 1719** | **4273 ± 1840** | **3442 ± 1992** | **1049 ± 1011** |

| ***Apis mellifera*** | | | | |
| --- | --- | --- | --- | --- |
| **Dorsal caput** | **Dorsal thorax** | **Dorsal waist** | **Dorsal abdomen** | **Ventral abdomen** |
| 1626 | 1681 | 2236 | 620 | 510 |
| 114 | 2363 | 2580 | 343 | 113 |
| 295 | 3471 | 1842 | 1037 | 221 |
| 1579 | 942 | 2245 | 279 | 1455 |
| 46 | 1078 | 1407 | 433 | 228 |
| 136 | 2077 | 4986 | 750 | 54 |
| 152 | 5831 | 6616 | 924 | 420 |
| 159 | 982 | 608 | 265 | 183 |
| 319 | 1641 | 1490 | 288 | 147 |
| 122 | 4404 | 2709 | 1541 | 38 |
| 110 | 2400 | 2486 | 447 | 102 |
| 234 | 1439 | 809 | 1135 | 129 |
| **408 ± 564** | **2359 ± 1505** | **2501 ± 1716** | **672 ± 411** | **300 ± 390** |
