# Supplementary material for: To be on the safe site – Ungroomed spots on the bee’s body and their importance for pollination
Source: PLoS One. 2017 Sep 6;12(9):e0182522. doi: 10.1371/journal.pone.0182522 (PMC5587100; doi:10.1371/journal.pone.0182522)
Supplement: S3 Table — (DOCX) [file pone.0182522.s005.docx]

**Supporting information**

**To be on the safe site – ungroomed spots on the bee’s body and their importance for pollination**

Laura Koch, Klaus Lunau & Petra Wester*

**S3 Table. Pollen grain amount of different body parts of *Apis mellifera* before and after grooming (raw data, and mean ± s.d.).**

| **Before grooming** | | | |
| --- | --- | --- | --- |
| **Dorsal** | **Ventral** | **Lateral right** | **Lateral left** |
| 2421 | 5980 | 3609 | 5334 |
| 3764 | 6269 | 2647 | 1197 |
| 1588 | 4998 | 3317 | 1312 |
| 3790 | 2843 | 3946 | 3874 |
| 3335 | 5525 | 5972 | 1412 |
| 2352 | 4562 | 1240 | 1682 |
| **2875 ± 892** | **5030 ± 1240** | **3455 ± 1561** | **2469 ± 1725** |
|  | | | |
| **After grooming** | | | |
| **Dorsal** | **Ventral** | **Lateral right** | **Lateral left** |
| 204 | 261 | 11 | 12 |
| 1283 | 140 | 28 | 25 |
| 248 | 3768 | 30 | 32 |
| 1669 | 2804 | 15 | 26 |
| 922 | 91 | 23 | 36 |
| 127 | 91 | 33 | 20 |
| 110 | 88 | 34 | 29 |
| 144 | 349 | 19 | 22 |
| 780 | 98 | 23 | 72 |
| 159 | 88 | 39 | 6 |
| **565 ± 565** | **778 ± 1344** | **26 ± 9** | **28 ± 18** |
