# Supplementary material for: To be on the safe site – Ungroomed spots on the bee’s body and their importance for pollination
Source: PLoS One. 2017 Sep 6;12(9):e0182522. doi: 10.1371/journal.pone.0182522 (PMC5587100; doi:10.1371/journal.pone.0182522)
Supplement: S4 Table — (DOCX) [file pone.0182522.s006.docx]

**Supporting information**

**To be on the safe site – ungroomed spots on the bee’s body and their importance for pollination**

Laura Koch, Klaus Lunau & Petra Wester*

**S4 Table. Time [min] spent grooming sunflower pollen and pine pollen of *Apis mellifera* and *Bombus terrestris* (raw data, mean ± s.d.).**

| ***Apis mellifera*** | | ***Bombus terrestris*** | |
| --- | --- | --- | --- |
| **Pine pollen** | **Sunflower pollen** | **Pine pollen** | **Sunflower pollen** |
| 6.583 | 2.400 | 2.350 | 4.483 |
| 4.200 | 4.333 | 3.583 | 13.167 |
| 4.533 | 10.683 | 3.167 | 9.083 |
| 2.683 | 11.250 | 0.700 | 4.950 |
| 0.167 | 13.750 | 3.250 | 9.50 |
| 11.250 | 16.600 | 4.000 | 0.833 |
| 5.717 | 15.283 | 15.383 | 5.567 |
| 20.517 | 17.433 | 5.200 | 5.500 |
| 21.583 | 21.650 | 2.633 | 2.967 |
| 5.950 | 26.200 | 3.333 | 10.617 |
| 10.317 | 10.650 | 3.367 | 4.767 |
| 9.517 | 23.250 | 4.067 | 5.533 |
| 9.050 | 10.383 | 3.817 | 9.600 |
| 9.150 | 28.867 | 4.483 | 19.333 |
| 24.450 |  | 5.183 | 19.300 |
| 13.633 |  | 2.900 |  |
| 19.567 |  | 7.667 |  |
|  |  | 3.950 |  |
|  |  | 1.400 |  |
|  |  | 4.483 |  |
|  |  | 7.200 |  |
|  |  | 4.800 |  |
|  |  | 1.350 |  |
|  |  | 9.117 |  |
|  |  | 5.133 |  |
|  |  | 3.117 |  |
|  |  | 5.500 |  |
|  |  | 4.383 |  |
|  |  | 3.883 |  |
| **10.522 ± 7.147** | **15.195 ± 7.771** | **4.462 ± 2.768** | **8.347 ± 5.483** |
