# Supplementary material for: To be on the safe site – Ungroomed spots on the bee’s body and their importance for pollination
Source: PLoS One. 2017 Sep 6;12(9):e0182522. doi: 10.1371/journal.pone.0182522 (PMC5587100; doi:10.1371/journal.pone.0182522)
Supplement: S5 Table — n = 20 bees per body part and control. Control animals were not stimulated. (DOCX) [file pone.0182522.s007.docx]

**Supporting information**

**To be on the safe site – ungroomed spots on the bee’s body and their importance for pollination**

Laura Koch, Klaus Lunau & Petra Wester*

**S5 Table. Amount of triggered grooming activities at the stimulated body parts and at unstimulated body parts of *Bombus terrestris*.** n=20 bees per body part and control. Control animals were not stimulated.

| **Body parts** | **Responses at the stimulated  body parts** | **Responses at the unstimulated body parts** |
| --- | --- | --- |
| Head | 16 | 1 |
| Antenna | 6 | 3 |
| Dorsal thorax | 1 | 5 |
| Wings | 0 | 3 |
| Dorsal waist | 0 | 3 |
| Dorsal abdomen | 1 | 5 |
| Ventral thorax | 5 | 0 |
| Ventral abdomen | 2 | 5 |
|  |  |  |
| **Control** | 8 |  |
