# Supplementary material for: To be on the safe site – Ungroomed spots on the bee’s body and their importance for pollination
Source: PLoS One. 2017 Sep 6;12(9):e0182522. doi: 10.1371/journal.pone.0182522 (PMC5587100; doi:10.1371/journal.pone.0182522)
Supplement: S6 Table — The sites are similar (s) to the location found in this study (Apis mellifera, Bombus terrestris) or different (d). References given in main text. (DOCX) [file pone.0182522.s008.docx]

**Supporting information**

**To be on the safe site – ungroomed spots on the bee’s body and their importance for pollination**

Laura Koch, Klaus Lunau & Petra Wester*

**S4 Table. Location of safe sites on the bees’ body after grooming behaviour following pollen contamination by pollen-sacs during visits of different plant species.** The sites are similar (s) to the location found in this study (*Apis mellifera*, *Bombus terrestris*) or different (d). References given in main text.

| **Bee species** | **Plant species [study area]** | **Safe site location** | **References** |
| --- | --- | --- | --- |
| *Apis mellifera* | *Pedicularis* L. spp. (Orobanchaceae) [China] | dorsal and ventral midline (s) | 38 |
| *Bombus* spp. | *Pedicularis* spp. [China and North America] | dorsal and ventral midline, especially the face, the dorsal midline of the thorax and abdomen, mainly anterior abdomen, and the anterior ventral abdomen (s) | 34,35,38 |
| *Bombus* spp. | *Chamaecrista fasciculata* (Michx.) Greene (Fabaceae) [North America] | midventral longitudinal line (s) | 41 |
| *Bombus* spp. | *Dodecatheon meadia* L. (Primulaceae), *Solanum dulcamara* L. (Solanaceae) [North America] | ventral anterior abdomen (s) | 32 |
| *Bombus* spp. | *Linaria* Mill., *Antirrhinum* L. (both Plantaginaceae), *Lobelia* L. spp. (Campanulaceae) [North America] | scutum (dorsal thorax between the wings and behind the head) and the frons (forehead) (s) | 33 |
| *Bombus* spp. | *Pedicularis* spp. [North America] | neck and the ventral thorax between the coxae (d) | 36,37 |
| *Bombus* spp. | *Astragalus* L. spp. (Fabaceae) [North America] | proboscidial fossa (groove on the underside of the head into which the proboscis folds) and the area between the head and the thorax (d) | 40 |
| *Bombus* spp. | diverse Rhinanthoideae (Orobanchaceae) [Europe] | cervical groove (neck) (d) | 49 |
| *Anthophora* Latreille 1803 (Anthophorinae, Apidae) | *Astragalus* spp. [North America] | proboscidial fossa and the area between the head and the thorax (d) | 40 |
| *Eucera* Scopoli 1770 (subgenus *Synhalonia* Patton 1879) (Anthophorinae, Apidae) | *Astragalus* spp. [North America] | proboscidial fossa and the area between the head and the thorax (d) | 40 |
| *Habropoda laboriosa* (Fabricius 1804) (Anthophorinae, Apidae) | *Vaccinium ashei* J.M. Reade (Ericaceae) [North America] | ventral head (d) | 39 |
| *Xylocopa violacea* (Linné 1758) (Xylocopinae, Apidae) | *Lathyrus latifolius* [Europe] | incision between head and thorax (d) | 52 |
| *Xylocopa* sp. Latreille 1802 | *Thunbergia grandiflora* Roxb. (Acanthaceae) [Europe] | dorsal thorax above the insertions of the legs and above the tegulae (s) | 26 |
| *Megachile ericetorum* (Lepeletier 1841) (Megachilinae, Megachilidae) | *Lathyrus latifolius* L. (Fabaceae) [Europe] | thorax especially above the tegulae (s) | 52 |
| *Euglossa imperialis* (Cockerell 1922) (Euglossini, Apinae, Apidae) | diverse plant spp. including orchids (containing pollinaria) [Central America] | on the anterior face and between the thorax and abdomen (s) neck and the gular region of the head (throat) (d) | 42 |
| diverse Euglossini | Marantaceae spp. [Latin America] | proboscidial fossa (d) | 51 |
| bees smaller than *Bombus* | *Pedicularis* spp. [North America] | ventral thorax between the coxae (d) | 36 |
| diverse bee species | *Viola* L. spp. (Violaceae) [Europe] | ventral parts of the head including the mandibular region, the ventral thorax and the basal joints of legs (d) | 31 |
| diverse bee species | two *Polyala* L. spp. (Polygalaceae) [Brasil] | on the left side of the ventral head (d) | 50 |
